# Inter-subject synchrony as an index of functional specialization in early childhood

**DOI:** 10.1038/s41598-018-20600-0

**Published:** 2018-02-02

**Authors:** Dustin Moraczewski, Gang Chen, Elizabeth Redcay

**Affiliations:** 1 Neuroscience and Cognitive Science Program, University of Maryland, College Park, MD 20742 USA; 2 Computation and Mathematics for Biological Networks, University of Maryland, College Park, MD 20742 USA; 3 Department of Psychology, University of Maryland, College Park, MD 20742 USA; 4 Scientific and Statistical Computing Core, National Institute of Mental Health, National Institutes of Health, Bethesda, USA

## Abstract

Early childhood is a time of significant change within multiple cognitive domains including social cognition, memory, executive function, and language; however, the corresponding neural changes remain poorly understood. This is likely due to the difficulty in acquiring artifact-free functional MRI data during complex task-based or unconstrained resting-state experiments in young children. In addition, task-based and resting state experiments may not capture dynamic real-world processing. Here we overcome both of these challenges through use of naturalistic viewing (i.e., passively watching a movie in the scanner) combined with inter-subject neural synchrony to examine functional specialization within 4- and 6-year old children. Using a novel and stringent crossed random effect statistical analysis, we find that children show more variable patterns of activation compared to adults, particularly within regions of the default mode network (DMN). In addition, we found partial evidence that child-to-adult synchrony increased as a function of age within a DMN region: the temporoparietal junction. Our results suggest age-related differences in functional brain organization within a cross-sectional sample during an ecologically valid context and demonstrate that neural synchrony during naturalistic viewing fMRI can be used to examine functional specialization during early childhood – a time when neural and cognitive systems are in flux.

## Introduction

Early childhood (roughly between the ages of 3 and 6) is marked by significant changes in multiple cognitive domains, including social cognition^1,2^, executive function^3^, attention^4^, memory^5,6^, and language^7^. However, a relatively small body of literature has used functional magnetic resonance imaging (fMRI) to examine the neural changes that occur in conjunction with these complex early behavioral and cognitive changes, leaving open the question of how functional brain development supports behavioral change.

**Figure 1:**
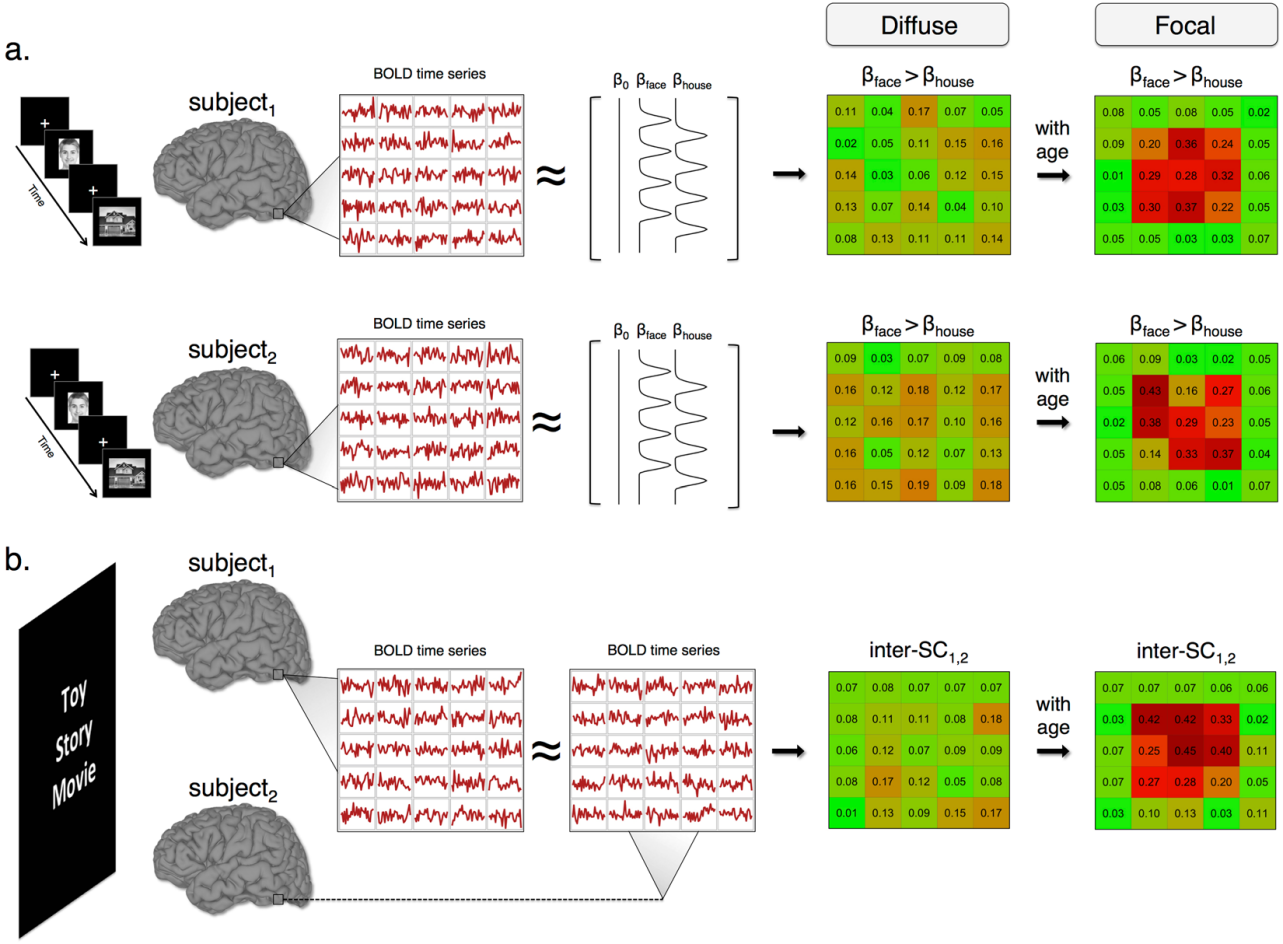
Functional specialization with inter-subject synchronization. (**a**) A schematic of how functional specialization is typically examined in task-based fMRI. Each subject participates in a task (e.g., viewing faces and houses). A GLM analysis reveals voxels whose activation is significantly greater for one condition compared to another (e.g., face > house). Functional specialization is then defined as weak and diffuse activation that becomes stronger and more focal with age. (**b**) A schematic of functional specialization during naturalistic viewing. Subjects view the same time-locked naturalistic stimulus in the scanner and, with age, patterns of activity become more focal and synchronous. Data in each schematic are simulated to conceptually illustrate our definition of functional specialization during (**a**) task and (**b**) naturalistic viewing. The face picture in (**a**) was decolorized and cropped. The original can be found at: https://commons.wikimedia.org/wiki/File:Face_–_Juno.jpg and is distributed under the CC BY-SA 4.0 license (https://creativecommons.org/licenses/by-sa/4.0/deed.en).

One dominant theory of functional brain development hinges on the concept of functional specialization (FS)^8^. Within this framework, as children’s behavioral and cognitive abilities mature, task-evoked neural activity will transition from topographically diffuse patterns of weak response amplitude toward a focal pattern of relatively stronger amplitude^8,9^. In this manner experience, behavior, and cognition form an interactive loop that effectively “tunes” the cortex for efficient processing^10^. For example, Fig. 1a provides a schematic of task-based FS for face processing within the fusiform gyrus of two participants. During childhood, cortical activation within the fusiform gyrus in response to faces is weak and diffuse. With age, response amplitude increases and activity patterns transition toward a more focal spatial topography^11–14^. While FS is often defined as a localized region whose selectivity increases with age for a specific computation and stimulus category, this framework is also compatible with network-wide task-evoked specialization^10^ as well as the specialization of intra-regional functional connectivity at rest^15,16^.

Here, functional specialization is distinct from functional specificity. We use the term specificity to refer to a significant neural response to one *specific* set of stimuli^17^ within a given brain region (e.g. the fusiform face area^18^), whereas the term specialization will refer to the *process* of developing neural specificity. Thus, a cortical region undergoes functional *specialization* in the event that it displays greater *specificity* for a cognitive process with age^8^. An example of FS can be seen in the role of the temporoparietal junction (TPJ) during theory of mind processing. In adults, the TPJ displays functional specificity during stories that require participants to consider the thoughts and intentions of other people^19^. Using a similar task in middle childhood, Gweon and colleagues found that response amplitude for stories that require theory of mind processing increases with age in the bilateral TPJ^20^. Thus, the TPJ exhibits FS for theory of mind processing; that is, specificity for theory of mind processing *increases* as a function of age within the TPJ. However, while FS has been demonstrated in older children during a variety of task-based experiments (e.g., theory of mind^20,21^, face processing^13,14^, attention^22^, episodic memory^23^) as well as network organization at rest^24–26^, less is known regarding this process in early childhood: a time of significant behavioral change.

**Figure 2:**
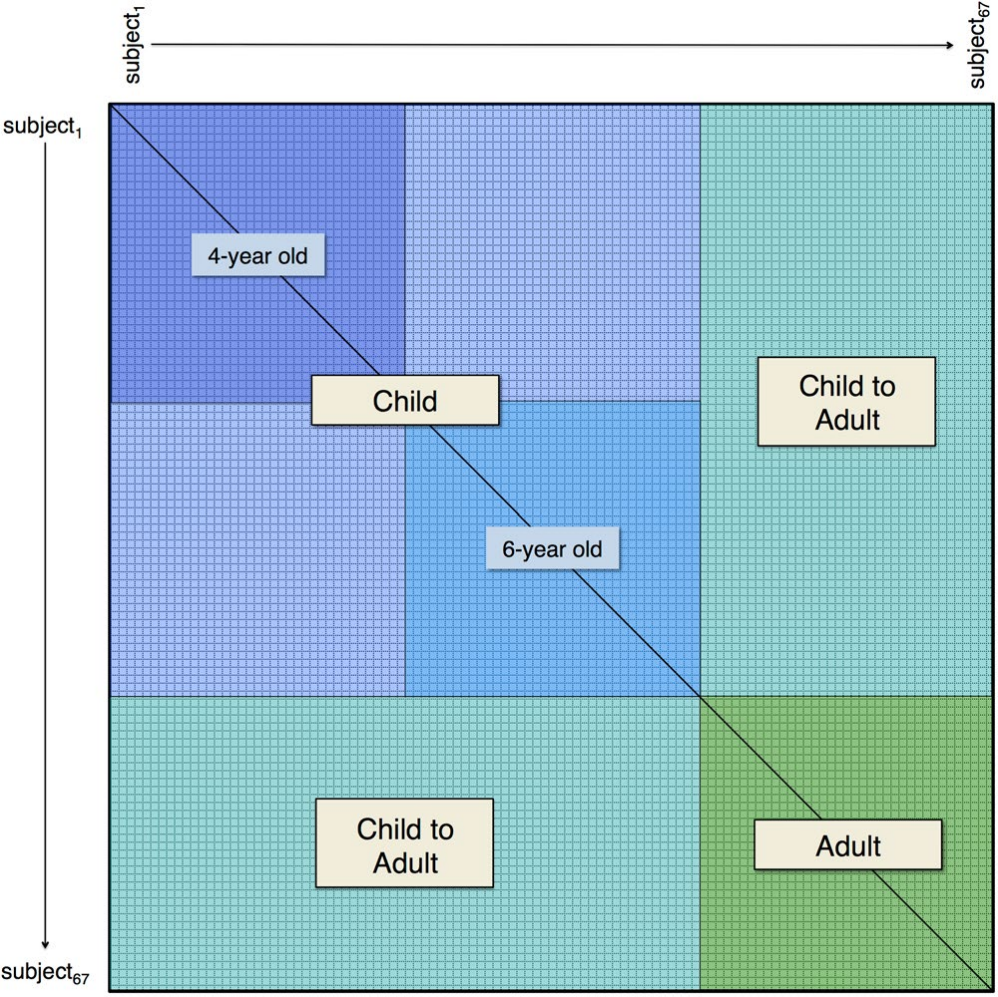
Pairwise inter-subject correlation matrix. A pairwise inter-SC matrix was calculated for each node on surface space. Each cell in this matrix contains a *Z*-transformed correlation between two subject’s BOLD signal time series.

This paucity of data likely results from the difficulty of acquiring artifact-free fMRI data from children in this age group. The instructions for task-based experiments are often too complicated for young children, whereas task-free resting state is unconstrained, which increases the risk of motion^27^. Studies in this age range primarily rely on imaging methods that are less susceptible to motion but also have limited spatial resolution such as functional near-infrared spectroscopy (fNIRS)^28–30^ and event-related potentials (ERP)^31,32^. The relatively few studies that have used functional MRI generally have to rely on very simplified designs for relatively short periods of time (see Raschle, *et al*.^33^ for a review). While this past work provides an emerging view of functional brain development during early childhood, these simplified fixed trial-based tasks may not capture the rich, dynamic processing inherent in real-world processing. Further, the discrepancy between simplified, trial-based tasks and real-world processing may be greatest in the case of social processing (cf. Risko *et al*.^34^), which undergoes dynamic changes during the preschool years^2^.

**Figure 3:**
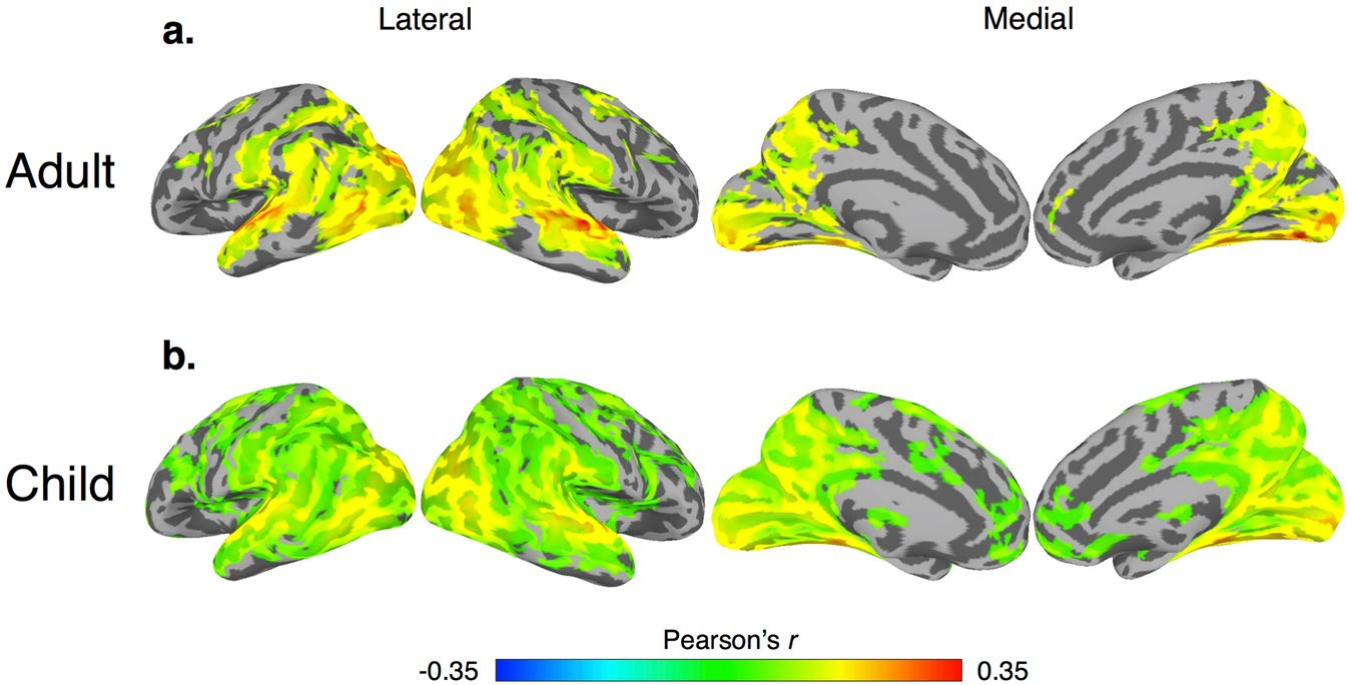
Within-group neural synchrony. Within-group inter-SC maps for the (**a**) Adult and (**b**) Child groups. Maps are thresholded at a nodewise *p* < 0.01 with a cluster extent of 315 mm^2^ to achieve FWE of *p* < 0.05.

Naturalistic viewing (i.e., the passive viewing of a movie) is a promising methodology to address these gaps in the literature because 1) it reduces the practical demands of a task-based fMRI experiment while also improving compliance^27^ and 2) is an ecologically valid method that is uniquely suited to probe the dynamic manner in which the brain processes complex social information^35–37^. Research in adults shows robust inter-subject synchrony^38,39^ with similarities to previous results from more constrained tasks^34^. This synchrony (assessed through a metric known as inter-subject correlation (inter-SC)) is thought to reflect a common mode of processing while subjects are viewing the same time-locked stimulus^40^ and can be used to decode functionally relevant aspects of a complex stimulus^38,41^. In addition, using event segmentation of naturalistic stimuli, previous work has shown that response amplitudes exhibit similar neural selectivity compared to task-based designs^36,42,43^. Thus, naturalistic viewing offers a promising method for understanding how the brain processes complex information and is ideally suited to probe FS in populations where complex task-based experiments may not be feasible (e.g., early childhood^27^ or in patient populations, such as autism^37^). In addition, while researchers have used movie viewing in young children to examine feasibility and motion^27^, math processing^44,45^, and intra-subject functional network organization^46^, no study has specifically investigated whole-brain FS using movie viewing methods in young children.

**Figure 4:**
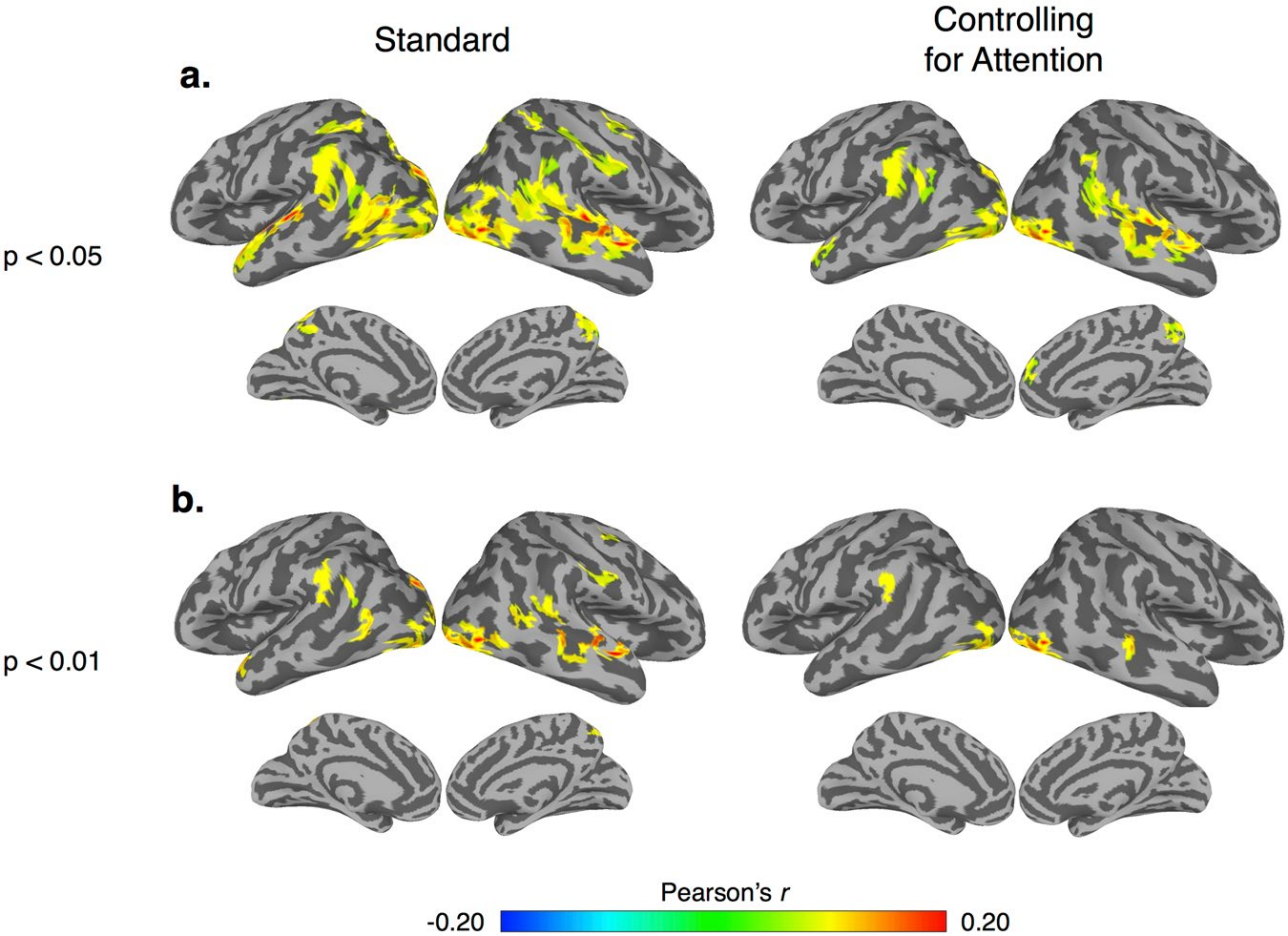
Adult > Child contrast. A between-group contrast of regions that exhibit greater inter-SC within the adult group for our standard preprocessing and controlling for attention with a (**a**) nodewise threshold of *p* < 0.05 with a cluster extent of 315 mm^2^ and (**b**) Nodewise threshold of *p* < 0.01 with a cluster extent of 150 mm^2^. All maps corrected to achieve FWE of *p* < 0.05.

In the current fMRI study, we use inter-SCs during naturalistic viewing as a method to probe FS in early childhood. Our reasoning is as follows: given that 1) FS predicts greater neural specificity with age^10^, 2) neural specificity can be seen during naturalistic viewing^36,42,43^, and 3) neural synchrony (inter-subject correlation) reflects common modes of processing to the same time-locked stimulus between individuals^40^, it follows that greater neural synchrony with age reflects FS for processing a complex, naturalistic stimulus (see schematic in Fig. 1b). In the current study we examine neural synchrony in a cross-section of 4- and 6-year old children, as well as in adults, while participants watched a clip of the movie Toy Story^47^. We chose the 4- and 6-year old age groups due to the extensive behavioral and cognitive changes that occur between these two ages. To probe age-related differences, we examined neural synchrony in two complementary ways: first we compared differences in within-group inter-SCs^48^, and second we examined age-related differences in child-to-adult inter-SCs (how similar the BOLD signal in each child is to the adult group, also known as neural maturity)^44^. We hypothesized that the Adult group would show greater neural synchrony compared to the Child group and that the response reliability between each child to the Adult group would increase with age. Rather than global whole-brain differences in synchrony between the two groups, we predicted that the movie-evoked responses would highlight FS within cortices associated with known behavioral and neural changes in this age range and which are engaged by the movie viewing. For example, given that the TPJ is implicated in studies of social-cognition, memory, language, and attention, we expected that FS would be seen in this region as watching an engaging movie requires such processing. In addition, to our knowledge this is the first developmental study to utilize a crossed random effect approach to linear mixed-effects modeling. This statistical method, which has been shown to more accurately control the false positive rate compared to other methods of group-level inter-SC analysis^48^, seeks to disentangle the violation of independence inherent in inter-SC data.

**Figure 5:**
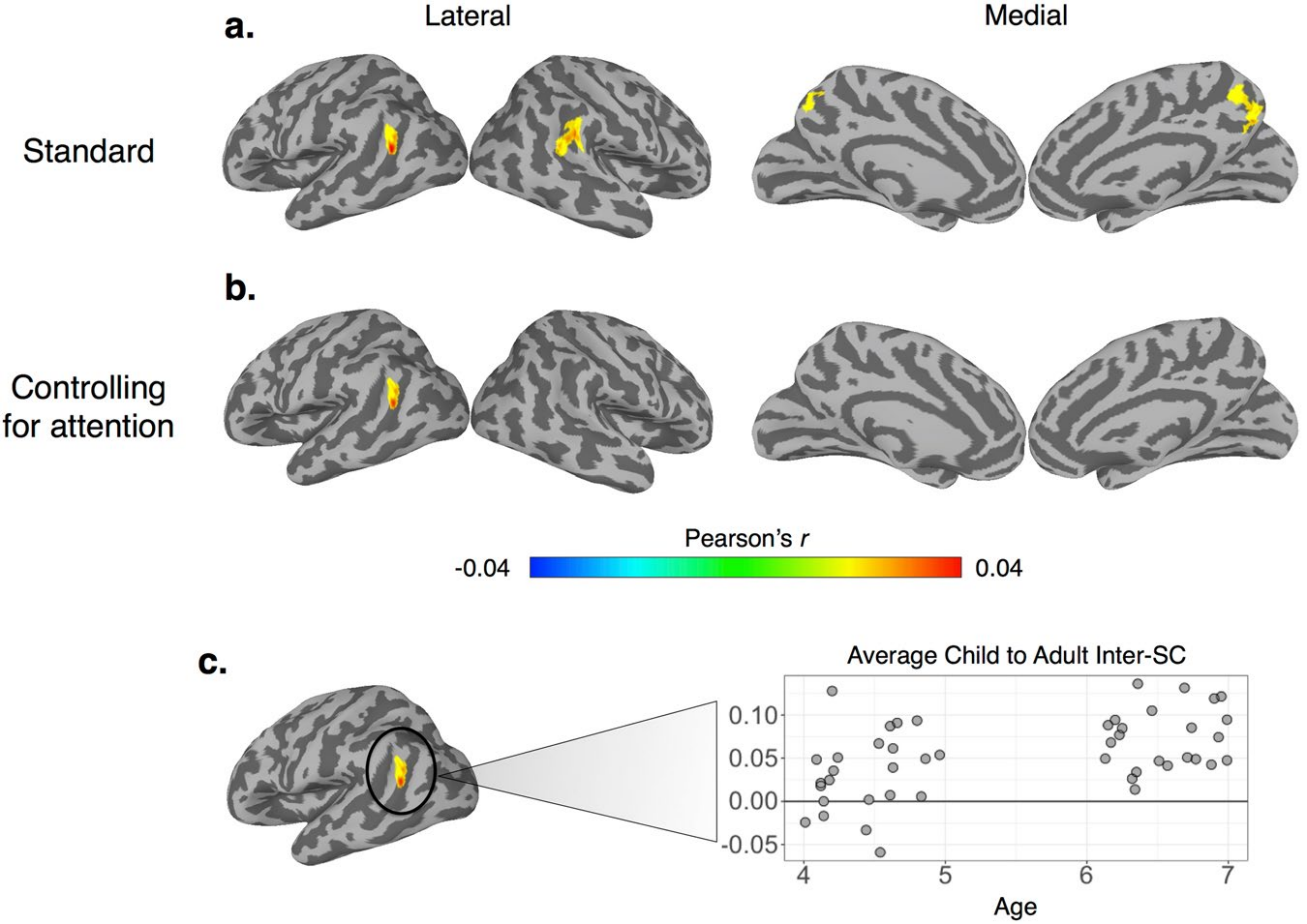
Relationship Child-to-Adult inter-SC and age. We use child age (in months) to predict Child-to-Adult inter-SC in our (**a**) Standard preprocessing and (**b**) controlling for attention. Maps are thresholded at nodewise *p* < 0.05 with a cluster extent of 150 mm^2^, uncorrected. (**c**) The average Child-to-Adult inter-SC within the left TPJ as a function of child age.

## Methods

### Participants

A cross-sectional sample of forty 4-year old children, thirty-eight 6-year old children, and twenty-four adults were recruited to participate in the study. All participants were native English speakers and had normal hearing, normal or corrected-to-normal vision, no history of neurological or psychological disorders, and no first-degree relatives with autism or schizophrenia as determined by self (adult participants) or parent (child participants) report. Children were recruited through the University of Maryland Infant and Child Studies database. All families were compensated monetarily and children were given a toy for their participation. Adult participants participated in exchange for either course credit or monetary compensation. With informed consent from adult participants and parents and assent from child participants, adults and children participated in two sessions: a behavioral battery and an MRI session. Ten 4-year old and two 6-year old children declined to participate in the MRI session and one 4-year old child was excluded due to a brain abnormality. After implementing a strict motion exclusion criterion (see fMRI preprocessing) the final sample was 67 (N = 23/23/21; 4 yr/6 yr/Adult). Within the final sample, full scale IQ did not statistically differ between the 4-year old, 6-year-old, and Adult groups (*F*(2,60) = 0.71, *p* = 0.49); however, IQ data were not available for 2 of the adult participants. See Table 1 for the participant demographics of the final sample. All protocols were approved by the University of Maryland Institutional Review Board and implemented in accordance with relevant guidelines and regulations.

### FMRI Stimuli

Functional data were acquired while participants passively-viewed a 6-minute, socially engaging clip (timestamp: approximately 26:28–32:28) from the movie Toy Story^47^. Participants were instructed to pay attention and watch the screen. The specific segment of the movie was chosen due to the social-cognitive demands placed on the viewer (e.g., to understand Buzz’s actions, the viewer needs to know that although Woody intended to roll a toy behind the dresser, Buzz is not aware of Woody’s actions even though the viewer knows the truth). All stimuli were presented using the Psychophysics Toolbox^49^ and viewed on a projection screen through a mirror mounted to the head coil. Sound was presented using Sensimetrics S14 insert earphones.

### Data Acquisition

MRI data were acquired using a Siemens 3T MAGNETOM Trio scanner using a 12-channel head coil. The imaging protocol consisted of a high-resolution T1-weighted magnetization prepared rapid gradient-echo (MPRAGE) image sequence (176 contiguous sagittal slices; 1 mm isotropic voxel size, TR/TE/inversion = 1900/2.52/900 ms; flip angle = 9°). Functional data consisted of 180 interleaved volumes of T2*-weighted echo planar images (EPI) (voxel size = 3 mm × 3 mm × 3.2 mm; TR/TE = 2000/24 ms; flip angle = 90°). The first 4 volumes of each EPI run were automatically discarded to allow for magnetization equilibrium.

**Figure 6:**
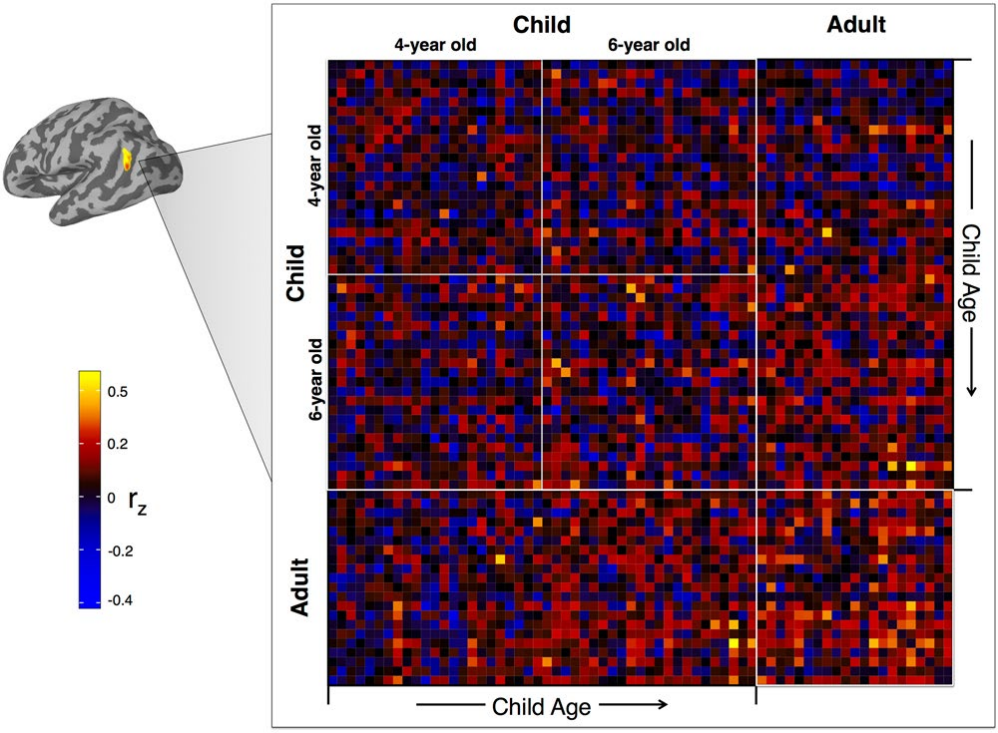
Pairwise inter-SC matrix within the left TPJ. We extracted all pairwise inter-SCs from the left TPJ cluster depicted in Fig. 5b. The Child group is arranged by child age such that older children are proximally closer to the adult portion of the matrix.

### Preprocessing

The anatomical T1-weighted images were processed using Freesurfer’s *recon-all* (version 5.1.0), an automated segmentation and surface construction algorithm^50^. Briefly, this algorithm uses voxel intensity to trace the border between tissue types (e.g., white matter and pial), which are then used to create a 2 dimensional mesh representation of the brain in original space along with segmented sub-cortical structures. To verify correct sub-cortical segmentation and surface reconstruction, two independent trained coders visually inspected the structural data, made edits to the white matter and pial surfaces, and reconstructed the segmentation and surfaces, if necessary.

Functional data were then preprocessed using a surface-based processing pipeline that utilized the Analysis of Functional Neuroimages (AFNI)^51^ and Surface Mapper (SUMA)^52^ software packages. Images were corrected for interleaved slice time acquisition and, together with the structural T1 and surface datasets, co-registered to a common functional base. Motion parameters created during this step were used to further exclude participants with excessive head motion. To quantify motion, we calculated the number of volumes that exceeded a framewise displacement (FD) of 0.5 mm, as calculated by $F{D}_{i}=|{\rm{\Delta }}{d}_{ix}|+|{\rm{\Delta }}{d}_{iy}|+|{\rm{\Delta }}{d}_{iz}|+|{\rm{\Delta }}{\alpha }_{ix}|+|{\rm{\Delta }}{\beta }_{ix}|+|{\rm{\Delta }}{\gamma }_{ix}|\,$^53^. Participants were then excluded based on a threshold that resulted in matched groups with respect to motion (final exclusion criteria consisted of runs where >9% of the functional volumes (i.e., 16 volumes) exceeded 0.5 mm in FD). After this exclusion, no statistically significant differences between mean frame displacement in the 4-year old (mean FD = 0.14 ± 0.07), 6-year old (mean FD = 0.16 ± 0.07), or the Adult groups (mean FD = 0.12 ± 0.05) (F(2,64) = 1.54, p = 0.22) or between the combined Child and the Adult groups (t(65) = 1.60, p = 0.12) were detected. In addition, to ensure that our results cannot be attributed to differences in micro-movements between the Child and Adult groups, we examined the number of volumes that exceeded a smaller motion threshold (0.15 mm) between the 4-year old (48.26 ± 41.33 volumes), 6-year old (47.26 ± 32.65 volumes), and Adult groups (46.86 ± 39.47 volumes). We found no statistically significant differences between groups (F(2,64) = 0.01, p = 0.99) or between the combined Child and Adult groups (t(65) = 0.09, p = 0.93). Further, we also provide consistent results from a stricter motion exclusion criterion (mean FD < 0.15 mm) in the Supplementary Information.

In lieu of 3D spatial normalization, white matter and pial surfaces were used as a mask to project the time series within gray matter voxels (using a mean mapping function) to a 2D standardized surface mesh (36,002 surface nodes per hemisphere) based on the participant’s own anatomy. We chose surface-based normalization to prevent bias induced through 3D spatial normalization of the children and adults into the same stereotaxic space^54^. Next, we normalized the signal intensity to a mean of 100 by scaling the time series by the nodewise average intensity. Individually defined white matter and lateral ventricle tissue masks from Freesurfer were resampled to the resolution of the volumetric functional data, eroded by one voxel in all directions (to prevent partial tissue overlap), and used to extract signal of no interest from the volumetric time series. The aforementioned tissue signal of no interest (white matter and ventricle), low-frequency linear, quadratic, and cubic trends, 0.01 to 0.1 hertz bandpass filter (in order to maintain temporal continuity during volume censoring), demeaned motion parameters, and their derivatives were applied in one nuisance regression. In addition, we employed a frame wise scrubbing procedure where functional volumes that exceeded 0.5 mm in translation and/or rotation were censored from the analysis^55^. Finally, the residuals from the nuisance regression were smoothed on a spherical surface mesh using a 7 mm Gaussian kernel. Previous work has shown that an ideal smoothing kernel for inter-subject correlation analysis is slightly larger than double the original voxel size^56^.

### Pairwise inter-Subject Correlation

Using the smoothed residuals from the nuisance regression, we then calculated all pairwise inter-SCs for each node on the surface mesh, as defined by ${r}_{i,j}={\rm{cor}}(t{s}_{i},t{s}_{j})$, where the correlation *r*_*i,j*_ equals the Pearson’s product-moment correlation between the time series, *ts*, of the *i*^*th*^ and *j*^*th*^ participant (*i* ≠ *j*). For group analysis, all pairwise inter-SCs for were Fischer z-transformed and entered into a symmetrical N x N matrix, where N = 67 (the total number of participants included in the final analysis). Thus, for each node on the surface mesh, we created a 67 × 67 pairwise inter-SC matrix for statistical analysis (Fig. 2).

### Statistical Analysis

Due to the fact that each participant’s time series data are represented *N* − 1 times in the correlation matrix (excluding the diagonal), many of the pairwise inter-SCs are correlated with each other. To properly account for this lack of independence, all statistical models were created using a crossed random effect structure^48^. For each statistical model, we predict inter-SC from model-dependent fixed effects while accounting for the random effects *of each* participant that contributes to the corresponding inter-SCs. Thus, the outcome measure *z*_*i*,*j*_ will include a random effect for the *i*^*th*^ and *j*^*th*^ participant, where *z*_*i,j*_ is the z-transformed inter-SC for the *i*^*th*^ and *j*^*th*^ participant. In addition, since the model is built using a symmetrical correlation matrix (excluding the diagonal), each outcome inter-SC will be represented twice, with an accompanying random effect for each participant that contributed to the correlation (i.e., both the top and bottom triangles of the correlation matrix are included in the analysis, see Fig. 2). This symmetrical redundancy allows the model matrix to consist of balanced and crossed random effects for each participant which allows for an accurate calculation of the shared variance between each effect. Using this crossed random effect structure to analyze pairwise inter-SCs has been shown to more effectively account for the violation of independence and control the false discovery rate compared to previous between-group inter-SC statistical methods^48^.

**Table 1:** Final sample participant demographics. Age, full scale IQ, and mean FD data are presented as mean (SD). *Full scale IQ data were not available for 2 participants in the Adult group. FD = frame displacement.

Group	*N*	Sex (M/F)	Age	Full Scale IQ	Mean FD
4-year old	23	9/14	4.44 (0.29)	115 (9.7)	0.14 (0.07)
6-year old	23	7/16	6.55 (0.30)	114 (13.7)	0.16 (0.07)
Adult	21	12/9	21.6 (2.94)	111* (9.6)	0.12 (0.05)

From the pairwise inter-SC matrix, multiple whole-brain tests and contrasts were performed. In order to determine which regions exhibited significant inter-SCs in response to the stimuli, separate within-group models were built for the 4-year old, 6-year old, Child (4- plus 6-year old groups), and Adult groups. For the within-group models, the intercept (group average inter-SC) was the effect of interest. Once the effects were calculated for each node on the surface, they were inverse Fischer z-transformed to the corresponding Pearson’s r for illustrative purposes. In addition, an Adult vs. Child between-group contrast was also calculated (for Adult vs 6-year old, Adult vs. 4-year old, and 6-year vs. 4-year old, see Supplementary Information). For the between-group contrast models, the effect of interest was group affiliation, while also accounting for crossed random effects. Each effect (intercept or group affiliation, respectively) was associated with a corresponding t statistic. To correct for multiple comparisons, we used 1000 Monte Carlo simulations to examine statistically significant clusters that could arise by chance. We first generated a volume of random functional data which we mapped to surface mesh using a mean mapping function and smoothed the results using a 7 mm FWHM Gaussian kernel. Due to our novel statistical methods, we present our results using multiple thresholds: nodewise *p* < 0.05 with a cluster extent of 315 mm^2^, and nodewise *p* < 0.01 with a cluster extent of 150 mm^2^, both of which correspond to a FWE of *p* < 0.05.

**Table 2:** Peak coordinates from within-group inter-SC maps.

Region	Left Hemisphere					Right Hemisphere				
	*r*	*t(20.47)*	*x*	*y*	*z*	*r*	*t(20.47)*	*x*	*y*	*z*
**a. Adult**										
Primary auditory	0.37	8.87	−56	−10	3	0.33	9.15	61	−8	−5
Extrastriate	0.32	7.10	−46	−75	11	0.26	7.63	39	−82	23
Primary visual	0.29	6.57	−15	−91	−15	0.30	6.29	5	−79	1
SPL	0.24	6.30	−13	−59	70	0.19	5.36	6	−71	52
STS	0.20	7.02	−57	−47	15	0.28	7.23	44	−37	0
FEF	0.16	5.08	−21	0	54	0.16	5.20	23	3	55
**b. Child**		*t(45.49)*					*t(45.49)*			
Primary auditory	0.24	7.23	−52	−17	4	0.21	7.44	57	−10	−1
Extrastriate	0.15	7.66	−39	−60	−1	0.20	9.30	37	−76	18
Primary visual	0.23	9.49	−3	−94	−8	0.22	8.00	16	−97	−1
SPL	0.12	7.24	−16	−54	66	0.09	5.52	10	−56	68
STS	0.09	5.89	−46	−51	21	0.19	7.19	51	−31	4
FEF	0.09	5.40	−31	−12	53	0.05	5.00	24	11	60

Note: All Pearson *r* values were normalized for group analysis and then inverse transformed (*z* to *r*) back into correlation values. All coordinates are in MNI space and were projected into volumetric space using a spatially normalized surface mesh. SPL, superior parietal lobule; STS, superior temporal sulcus; FEF, frontal eye field.

### Controlling for Attention

Since it is possible that the observed group differences could be a result of systematic group differences in attention, we conducted a separate analysis where we used the average BOLD signal within a frontal eye field (FEF) region of interest (ROI) as a nuisance regressor of no interest. Since the FEF has been implicated in top-down attention and saccadic eye movements^57^, we used the activity within this region as a proxy for individual differences in attention (cf. Redcay *et al*.^58^). To create the ROI, we utilized Neurosynth, a meta-analytic tool that amalgamates the fMRI results from thousands of published studies^59^. We exported a statistical map that corresponded to the search term ‘saccade’, thresholded at a voxelwise threshold of p < 0.01, and found the peak effect estimate that corresponded to the FEF (MNI coordinate: 28, −4, 52). Using this coordinate, we identified the corresponding node on the surface mesh and extended the region 8 nodes in all directions. Then, we preprocessed each participant’s functional data using the same steps as above, while also including each individual’s mean signal within the FEF ROI in the first-level nuisance regression. These residuals were then smoothed using a 7 mm Gaussian kernel. Finally, all pairwise inter-SCs were calculated on the FEF-regressed functional data in the same manner as above and the same group-wise statistical tests and contrasts were made.

**Table 3:** Peak coordinates from Adult versus Child groups.

Region	Left Hemisphere					Right Hemisphere				
	*r*	*t(49.89)*	*x*	*y*	*z*	*r*	*t(49.89)*	*x*	*y*	*z*
**a. Standard**										
TPJ	0.12	3.48	−59	−49	16	0.12	3.67	54	−46	13
STS	0.08	2.40	−57	−53	9	0.17	4.89	51	−37	−3
pSTS	0.06	2.59	−40	−54	12	0.13	2.69	54	−36	7
Extrastriate	0.18	3.55	−46	−75	11	0.13	3.69	47	−72	−8
Superior parietal	0.18	4.83	−13	−59	70	0.14	4.70	7	−72	51
**b. Controlling for Attention**										
TPJ	0.07	2.84	−49	−58	24	0.09	3.66	37	−57	15
STS	0.11	3.09	−53	−20	0	0.15	4.79	51	−37	−3
pSTS	0.11	2.37	−50	−46	8	0.09	2.60	50	−42	12
Extrastriate	0.10	2.99	−55	−61	8	0.14	3.98	47	−80	−9

Note: All Pearson *r* values were normalized for group analysis and then inverse transformed (*z* to *r*) back into correlation values. All coordinates are in MNI space and were projected into volumetric space using a spatially normalized surface mesh. TPJ, temporoparietal junction; STS, superior temporal sulcus; pSTS posterior superior temporal sulcus; dmPFC, dorsal medial prefrontal cortex.

One assumption of this method is that each group is relatively homogenous in FEF response. To ensure that our observed results cannot be attributed to outliers in the signal within the FEF (a proxy for an attentional outlier – that is, a participant who may be viewing the movie in a systematically different way compared to the rest of their group), we examined activity within this ROI for outliers. This analysis yielded five children who were outliers in FEF inter-SC (see Supplementary Methods and Supplementary Figure S4a). However, our main findings remain consistent after we exclude these outliers (Supplementary Figure S4b,c).

**Table 4:** Peak coordinates of the relationship between child-to-adult inter-SC and age.

Region	Left Hemisphere					Right Hemisphere				
	*r*	*t(62.13)*	*x*	*y*	*z*	*r*	*t(62.13)*	*x*	*y*	*z*
TPJ	0.03	3.15	−47	−55	20	0.03	3.44	55	−45	25
Precuneus	0.03	2.08	−12	−53	57	0.03	2.25	4	−51	61

Note: All Pearson *r* values were normalized for group analysis and then inverse transformed (*z* to *r*) back into correlation values. All coordinates are in MNI space and were projected into volumetric space using a spatially normalized surface mesh. TPJ, temporoparietal junction; STS, superior temporal sulcus; pSTS posterior superior temporal sulcus; dmPFC, dorsal medial prefrontal cortex.

### Age-related Differences in Inter-SC

To examine age-related differences in inter-SC, we used child-to-adult inter-SC (also known as neural maturity^44^). This measure indexes how the BOLD signal within each child participant correlates with each adult participant (cyan portion of Fig. 2). Using these inter-SCs, we constructed a crossed random effect model where child age (in months) predicted Child-to-Adult inter-SC. All whole-brain maps are presented using a node-wise threshold of *p* < 0.05 with a cluster extent threshold of 150 mm^2^, uncorrected. It should be noted that current cluster-based methods to correct for multiple comparisons have not been assessed using child-to-adult inter-SC or crossed random effect analysis. In addition, we provide a traditional analysis of the relationship between Child-to-Adult inter-SC and age in the Supplementary Information.

### Data Availability

The datasets generated during and analyzed during the current study are available from the corresponding author on reasonable request.

## Results

### Adults show greater inter-SC compared to children

To examine group-wise differences in neural synchrony, we first examined which brain regions exhibited statistically significant inter-SC within each group separately. We generated two whole-brain models with crossed random effects using the following subsets of the pairwise inter-SC matrix (Fig. 2): Adult (green matrix) and Child (all blue matrix) (for within-group maps of the 4- and 6-year old groups see Supplementary Figure S1a,b). Consistent with previous work^37–39^, all within-group models exhibited statistically significant inter-SC across much of the occipital, temporal, and parietal cortices, with superior and inferior temporal, extrastriate, and occipital areas showing the strongest synchrony – a pattern that reflects the perception of complex visual and auditory stimuli including language and animated agents (Fig. 3a,b - Adult and Child, respectively, Supplementary Figure S1a,b- 4- and 6-year old groups, respectively, see Table 2a,b and Supplementary Table S1a,b for corresponding peak coordinates).

To examine group differences, we then constructed a crossed random effect contrast model. For the Adult vs. Child model (green vs. large blue matrix, Fig. 2), the Adult group exhibited significantly stronger inter-SC within the bilateral temporoparietal junction, middle and superior temporal, extrastriate, and superior parietal (Fig. 4, see Table 3 for peak coordinates) (see Supplementary Figures S2a–c for Adult vs. 6-year, Adult vs. 4-year, and 6 vs. 4-year old group contrasts, respectively). To test whether the Adult vs. Child results could be attributed to systematic differences in top-down attention or saccadic eye movements between the two groups, we also constructed a separate model using the functional data where the BOLD signal from the FEF was included in the nuisance regression. After using the signal within the FEF as a proxy of top-down attention and saccadic eye movements, the Adult group continued to exhibit stronger inter-SC in the bilateral temporoparietal junction, middle and superior temporal, dorsal medial prefrontal, and extrastriate cortices compared to the Child group (Fig. 4, see Table 3b for peak coordinates). In addition, these results cannot be attributed to micro-differences in motion between the two groups (Supplementary Figure S3a,b).

### Inter-SC in the temporoparietal junction becomes more adult-like with age

We then examined the relationship between child age (in months) and individual differences in inter-SC. For the individual differences whole-brain models, we used the Child-to-Adult inter-SCs that reflect the degree to which each child participant correlates to each adult participant (cyan matrix, Fig. 2)^44^. We found no clusters that met statistical significance after a stringent correction for multiple comparisons. However, since crossed random effect models have not been used to test the relationship between child-to-adult inter-SC and a covariate (e.g., age), we also provide our results using a more liberal cluster-based correction (nodewise p < 0.05 with a cluster extent of 150 mm^2^, uncorrected). In the whole-brain model that used age to predict Child-to-Adult inter-SC, we found that activity in the bilateral temporoparietal junction and dorsal precuneus became more similar to the Adult group with age (Fig. 5a, see Table 4 for peak coordinates). After controlling for differences in attention, only the left temporoparietal region survived (Fig. 5b, peak r: 0.03, t(62.13) = 3.28, MNI coordinate: −47 −55 20). In addition, we show consistent results using a standard statistical analysis of child-to-adult inter-SC (see Supplementary Information). In addition, using the average inter-SCs within the left TPJ cluster in Fig. 5b, we show average Child-to-Adult inter-SC (for each child) as a function of child age in Fig. 5c. Finally, we also extracted the pairwise inter-SC matrix to present for illustrative purposes in Fig. 6. This figure highlights the pairwise inter-SCs that were used in previous models: within-group, between-group, and Child-to-Adult inter-SC.

## Discussion

The current study examined functional specialization (FS) through the lens of inter-subject neural synchrony during naturalistic viewing in early childhood. We found that the Child group exhibited significantly less inter-SCs in the bilateral TPJ, superior temporal sulcus (STS), superior temporal gyrus (STG), precuneus, as well as extrastriate and visual areas compared to the Adult group – a pattern that held after controlling for a proxy of top-down attention and saccadic eye movements (through the inclusion of signal within the frontal eye field (FEF) in the nuisance regression) and micro-movements (see Supplementary Information). In addition, Child-to-Adult inter-SCs, an index of individual similarity between a child participant and the adult group, increased as a function of age in the TPJ. However, this result did not survive a stringent correction for multiple comparisons. Our results highlight age-related differences in functional brain organization within a cross-sectional sample during early childhood – a time where task-based and resting-state fMRI data are difficult to obtain due to task demands and/or motion artifact^27^, respectively. In addition, the current study is the first developmental application of a crossed random effects inter-SC analysis – a novel statistical approach that properly controls the false positive rate relative to previous group-level inter-SC analysis methods^48^.

We examined FS through two distinct metrics: within-group inter-SCs and child-to-adult inter-SCs. The first metric, within-group inter-SCs, tests the degree to which an individual’s neural pattern deviates from the group’s mean response. Less inter-SC within the child group is evidence of weak and diffuse patterns of functional activation whereas focal activation patterns begets greater inter-subject synchrony (schematic in Fig. 1b). In the current study, we observed less within-group inter-SC in the Child group compared to the Adult group. This reduced inter-SC suggests reduced functional specialization to stimuli during naturalistic movie viewing and is consistent with the diffuse-to-focal functional trajectory posited by the Interactive Specialization hypothesis of functional brain development^10^. These results also suggest that adults recruit more common neural resources while processing naturalistic stimuli whereas children may utilize more variable and individualized neural resources. Similar findings have been shown in older adults, such that younger adults exhibiter greater inter-SC compared to older adults^60^. Taken together with the current study, these findings suggest that neural synchrony during naturalistic viewing may occur along an inverted-U shaped curve. Importantly however, the group differences in the current study are not global. Rather, the Adult group shows greater inter-SC in regions topographically similar to the Default Mode Network (DMN)^61,62^, as well as regions within the extrastriate cortices.

Our second index of FS was child-to-adult inter-SC^44^ - specifically how the synchrony between each child and each adult (Fig. 2, cyan portion of inter-SC matrix) varied as a function of child age. While our results are non-statistically significant after a stringent correction for multiple comparisons, we found some evidence that as children age response patterns within the bilateral TPJ and dorsal precuneus become more ‘adult-like’. However, after controlling for a proxy of top-down attention and saccadic eye movements through regressing out signal within the FEF, we find that only response in the left TPJ maintains this relationship with age. The disappearance of the right TPJ and precuneus likely speaks to the role that these regions play in complex attentional processes^63,64^. It is also worth noting that the signal within the FEF is not an absolute control for attention. Future studies should integrate behavior and neural metrics of attention to examine its effect on neural synchrony during naturalistic viewing.

The TPJ is a functionally heterogeneous region that is implicated in attention, memory, and theory of mind processing^64,65^. In addition, the TPJ exhibits FS for mental state representation in older children^20,21^ and this specialization is related to children’s improving explicit social-cognitive abilities^20^. Thus, we speculate that greater inter-SC within the TPJ during naturalistic viewing may be indicative of FS for social processing as watching a movie requires the viewer to engage in such processing. For example, to follow the plot of a movie a viewer must identify intentional agents from the background and manage the multiple representations of characters’ mental states. However, given that the TPJ is also implicated in attention orienting, language, and memory, the current study cannot directly disentangle the relative contribution of social processing to age-related differences in child-to-adult inter-SCs. Future studies should seek to relate child-to-adult inter-SCs to these developing social and cognitive abilities in order to elucidate the functional role of left TPJ FS in early childhood. Our results are consistent with recent cross-sectional and longitudinal work that suggests the left TPJ becomes more functionally connected at the whole-brain level during rest between the ages of 3 and 6 years old^66,67^. However, the current cross-sectional nature of the current study cannot speak directly to an age-related trajectory of FS during naturalistic viewing. Future studies should incorporate larger and longitudinal samples to investigate FS during naturalistic viewing through child-to-adult inter-SC in order to have the power to detect age-related effects within this narrow age range.

The two metrics of FS discussed here (within-group and child-to-adult) offer different, though complementary windows into functional brain development during early childhood. While the difference in within-group neural synchrony provides differences *between* early childhood and adulthood, the child-to-adult metric examines age-related individual differences *within* early childhood. We find convergence between these two methods within the response patterns of the bilateral TPJ and dorsal precuneus though, as described above, after controlling for a proxy of attention, only the left TPJ remains. While we do find convergence between methods, we also note the differences. The extent of the Adult >Child within-group contrast (Fig. 4) is much greater than that of the relationship between child-to-adult inter-SCs and age (Fig. 5). These findings suggest FS within regions of the default mode between childhood and adulthood with the left TPJ in particular showing FS in early childhood. Future studies should examine child-to-adult inter-SC during naturalistic viewing throughout all phases of development to obtain a more continuous measure of age-related differences.

The regions that exhibit greater neural synchrony within the Adult group are broadly topographically similar to the DMN. The DMN contains regions such as the TPJ, dmPFC, precuneus, inferior frontal gyrus (iFG), and middle temporal cortices, and has been implicated in social-cognitive processing, memory, and language^68–70^. In addition, these regions are also topographically similar to those attributed to long timescale processing^71–73^. In adults, temporal manipulation of naturalistic stimuli has uncovered a cortical hierarchy where certain regions (e.g., primary sensory cortices) exhibit significant neural synchrony to both an intact movie and a scrambled movie whereas other regions (e.g., those associated with the DMN) only exhibit significant synchrony to information presented over long timescales, such that synchrony is only seen in the intact and not the scrambled movie^71,74^. Neural synchrony to information presented over long timescales is thought to reflect a cortical memory where the brain integrates incoming perceptual information with one’s internal representation of the unfolding plot^75^. We previously discussed our results in terms of the development of multiple cognitive and neural systems (e.g., social-cognitive, memory, attention, language), but these age-related differences may not be related to development of these systems *per se* but are indicative of an immature cortical hierarchy for processing information at long timescales. Thus, perhaps the cortex specializes with age for a domain-general temporal hierarchy that enables more efficient integration of information over multiple timescales. However, the current study cannot address this hypothesis.

In conclusion, the current study utilizes naturalistic viewing, neural synchrony, and a novel approach to mixed-effects modeling to examine functional specialization during early childhood. We use complimentary metrics of neural synchrony to show 1) brain regions that exhibit more variable and individualized response patterns in young children compared to adults and 2) that response in the left TPJ becomes more ‘adult-like’ as a function of age. Our results provide an ecologically valid and novel perspective on functional specialization in early childhood – a time of rapid cognitive and neural development.

## Electronic supplementary material

Supplementary Information
