# Supplementary material for: Inter-subject synchrony as an index of functional specialization in early childhood
Source: Sci Rep. 2018 Feb 2;8:2252. doi: 10.1038/s41598-018-20600-0 (PMC5797124; doi:10.1038/s41598-018-20600-0)
Supplement: Supplementary file 1 — Supplementary Information [file 41598_2018_20600_MOESM1_ESM.pdf]

**Inter-subject synchrony as an index of functional specialization in early childhood**

Dustin Moraczewski[1,2,3] Gang Chen[4] & Elizabeth Redcay[1,3]

[1] Neuroscience and Cognitive Science Program, University of Maryland, College Park, MD, 20742

[2] Computation and Mathematics for Biological Networks, University of Maryland, College Park, MD, 20742

[3] Department of Psychology, University of Maryland, College Park, MD, 20742

[4] Scientific and Statistical Computing Core, National Institute of Mental Health, National Institutes of Health, USA

**Supplementary Materials**

## Methods

### Low-motion sample

To ensure that our Adult versus Child group comparisons cannot be attributed to differences in micro-movements between groups, we also provide result from a low-motion sample in which we excluded all participants with mean frame displacement (FD) > 0.15mm. Our sample size after this exclusion was $N$ = 40 (16 Adults; 24 Children). There were no statistical difference between mean FD between the Adult and Child groups (t(38)=0.90, p=0.38; mean FD (in mm) Adult = 0.10 ± 0.03, mean FD Child = 0.09 ± 0.03) or the 4- and 6-year old groups (t(16)=1.08, p=0.30). We then constructed an identical crossed random effect model[1] to predict within-group inter-subject correlation (inter-SC) from group affiliation. As in the main text, we constructed two models: one using a standard inter-SC and one controlling for a proxy of top-down attention and saccadic eye movements through including the signal with the frontal eye field (FEF) in the first-level nuisance regression (see Methods). Results are provide in Supplementary Figure 3a,b with corresponding coordinates of the peak inter-SC estimates in Supplementary Table 3a,b. Due to the reduction in power given the small sample size, we provide our results with a nodewise $p < 0.05$ with a cluster extent of 150mm$^2$, uncorrected.

### Detection of attentional outliers

It is possible that our between-group results could be attributed to systematic differences in attention between groups. To address this concern, we provide an identical analysis in the main manuscript where we control for a proxy of top-down attention and saccadic eye movements: activity within the FEF. A second concern is that one assumption of a between-group test is that the individuals within each group serve as a relatively homogenous and representative sample of the group to which they are affiliated. By examining inter-SC within the FEF timeseries we can identify outlier individuals, suggesting that they may be viewing the movie in a qualitatively different way.

We examined within-group inter-SC within each participant's FEF (see Methods in the main text for ROI definition). Using the mean time series within the FEF, we conducted a leave-one-out inter-SC analysis to examine how each participant correlates to the average of the rest of their own group (Supplementary Figure S4a), which we then compared between the Child and Adult groups. We found that the Adult group exhibited higher inter-SC (mean±sd: 0.53±0.12) compared to the Child group (0.41±0.18) ($t(56)=3.18$, $p<0.01$) (Figure 4Sa). To define possible attentional outliers, we used the median absolute deviation, which is less resistant to outliers compared to the mean. We detected five outliers within the Child group (three 4-year and two 6-year olds) (Supplementary Figure S4a, dashed red oval) and no outliers in the adult group. With the attentional outliers within the Child group removed, the Adult group still exhibited higher inter-SC compared to the Child group ($t(35)=2.20$, $p<0.05$).

Thus, our data suggest that there may be a systematic attentional difference between the two groups, which reinforces our use of the signal within the FEF as a covariate.

To ensure that our main findings are being driven by the attentional outliers identified in the Child group, we removed these outliers and conducted Adult vs. Child within-group (Supplementary Figure S4b) and a Child-to-Adult (Supplementary Figure S4c) inter-SC crossed random effect models on our Standard preprocessed data, as described in the main text. Results from this analysis are generally consistent with the analysis reported in the manuscript. Of note, however, is that the relationship between Child-to-Adult inter-SC and age (in months) highlights more regions than in our main analysis (e.g., superior temporal sulcus and posterior parietal areas). These differences could be the result of removing the attentional outliers (thus the group is more homogenous in terms of our proxy for attention) and/or a result of our lower power to detect individual differences due to the removal of five data points. However, the bilateral TPJ and precuneus regions from the analysis in the main paper (Figure 5a, main text) remain even after the removal of these outliers.

**Standard analysis of child-to-adult inter-SC**

Since crossed random effect models have not been used to investigate individual differences in inter-SC, we also provide a standard analysis of child-to-adult inter-SC (also known as neural maturity[2]). Using the Child-to-Adult portion of pairwise inter-SC matrix (cyan portion of Figure 2 in the main text), we

calculated the average inter-SC for each child. This metric reflects the average correlation of a given child to each adult. This averaging procedure yielded one inter-SC value per node on surface mesh per child. We then correlated inter-SC with child age (in months). We provide results from a node-wise threshold of $p < 0.05$ to directly compare to the crossed random effect model in the main text and a more stringent nodewise threshold of $p < 0.01$ with a cluster correction of $k = 150mm^2$ to reflect a FWE of $p < 0.05$ (Standard preprocessing, Supplementary Figure 5a,b, peak coordinates: Supplementary Table 4a,b; Controlling for Attention, Supplementary Figure 4a,b, peak coordinates: Supplementary Table 5a,b).

## Supplementary References


1. Chen, G., Taylor, P. A., Shin, Y. W., Reynolds, R. C. & Cox, R. W. Untangling the relatedness among correlations, part II: inter-subject correlation group analysis through linear mixed-effects modeling. *Neuroimage* **147,** 825-840 (2016).
2. Cantlon, J. F. & Li, R. Neural activity during natural viewing of Sesame Street statistically predicts test scores in early childhood. *PLoS Biol.* **11,** e1001462; doi:10.1371/journal.pbio.1001462.g005 (2013).

| | Left Hemisphere | | | | | Right Hemisphere | | | | |
|---|---|---|---|---|---|---|---|---|---|---|
| Region | *r* | *t*(22.47) | *x* | *y* | *z* | *r* | *t*(22.47) | *x* | *y* | *z* |
| **a. Six** | | | | | | | | | | |
| Primary auditory | 0.30 | 6.55 | -52 | -17 | 4 | 0.27 | 6.63 | 62 | -8 | -6 |
| Extrastriate | 0.19 | 4.78 | -44 | -79 | 12 | 0.18 | 5.62 | 42 | -83 | 21 |
| Primary visual | 0.30 | 7.85 | -3 | -94 | -11 | 0.23 | 5.31 | 16 | -97 | -1 |
| SPL | 0.11 | 3.74 | -17 | -56 | 71 | 0.11 | 3.65 | 7 | -53 | 70 |
| STS | 0.22 | 5.21 | -52 | -36 | 9 | 0.19 | 4.90 | 44 | -40 | 1 |
| FEF | 0.11 | 4.04 | -32 | -12 | 52 | 0.08 | 3.25 | 29 | -6 | 64 |
| **b. Four** | | *t*(22.47) | | | | | *t*(22.47) | | | |
| Primary auditory | 0.20 | 3.84 | -53 | -13 | 4 | 0.20 | 4.93 | 55 | -9 | 3 |
| Extrastriate | 0.19 | 5.18 | -44 | -73 | 10 | 0.20 | 7.03 | 39 | -75 | 10 |
| Primary visual | 0.20 | 5.42 | 0 | -91 | -6 | 0.26 | 6.91 | 8 | -91 | -1 |
| SPL | 0.12 | 4.48 | -30 | -47 | 56 | 0.11 | 4.00 | 12 | -61 | 69 |
| STS | 0.13 | 3.80 | -54 | -44 | 12 | 0.16 | 4.33 | 49 | -30 | 3 |
| FEF | 0.10 | 3.70 | -25 | -10 | 63 | 0.08 | 4.33 | 21 | 9 | 58 |

**Supplementary Table S1:** *Peak coordinates from within-group Child inter-SC maps from Supplementary Figure S1.*

Note: All Pearson *r* values were normalized for group analysis and then inverse transformed (*z* to *r*) back into correlation values. All coordinates are in MNI space and were projected into volumetric space using a spatially normalized surface mesh. SPL, superior parietal lobule; STS, superior temporal sulcus; FEF, frontal eye field.

| | Region | Left Hemisphere *r* | *t*(30.41) | *x* | *y* | *z* | Right Hemisphere *r* | *t*(30.41) | *x* | *y* | *z* |
|---|---|---|---|---|---|---|---|---|---|---|---|
| **a. Adult > Six** | | | | | | | | | | | |
| | Primary Visual | 0.18 | 3.84 | -27 | -92 | 18 | 0.18 | 3.49 | 48 | -78 | -6 |
| | pSTS | - | - | - | - | - | 0.12 | 2.81 | 50 | -42 | 12 |
| | mSTS | - | - | - | - | - | 0.14 | 3.03 | 51 | -37 | -3 |
| | Extrastriate | - | - | - | - | - | 0.14 | 2.90 | 54 | -62 | 0 |
| | TPJ | 0.11 | 2.23 | -57 | -48 | 33 | - | - | - | - | - |
| | SPL | 0.19 | 3.99 | -13 | -59 | 70 | 0.15 | 2.7 | 7 | -64 | 65 |
| **b. Adult > Four** | | | *t*(30.41) | | | | | *t*(30.41) | | | |
| | Primary Visual | 0.15 | 4.20 | -32 | -85 | -7 | 0.20 | 5.07 | 39 | -84 | -15 |
| | pSTS | 0.14 | 3.91 | -61 | -49 | 15 | 0.12 | 2.93 | 52 | -48 | 4 |
| | mSTS | 0.27 | 3.63 | -63 | -28 | 7 | 0.25 | 3.90 | 61 | -32 | 4 |
| | Extrastriate | 0.19 | 3.68 | -50 | -68 | 8 | 0.10 | 2.79 | 39 | -79 | 12 |
| | TPJ | 0.14 | 3.91 | -61 | -49 | 15 | 0.12 | 2.63 | 54 | -46 | 13 |
| | SPL | 0.12 | 3.54 | -33 | -53 | 54 | 0.14 | 3.27 | 43 | -48 | 57 |
| | Precuneus | 0.12 | 3.41 | -10 | -44 | 54 | 0.14 | 2.79 | 9 | -58 | 54 |
| **c. Six > Four** | | | *t*(31.79) | | | | | *t*(31.79) | | | |
| | mSTS | - | - | - | - | - | 0.13 | 2.55 | 51 | -36 | 6 |

**Supplementary Table S2:** *Peak coordinates from between group contrast maps from Supplementary Figure S2.*

Note: All Pearson *r* values were normalized for group analysis and then inverse transformed (*z* to *r*) back into correlation values. All coordinates are in MNI space and were projected into volumetric space using a spatially normalized surface mesh. pSTS, posterior superior temporal sulcus; mSTS, middle superior temporal sulcus, TPJ, temporoparietal junction; SPL, superior parietal lobule.

| | Region | Left Hemisphere $r$ | $t_{(28.12)}$ | $x$ | $y$ | $z$ | Right Hemisphere $r$ | $t_{(28.12)}$ | $x$ | $y$ | $z$ |
|---|---|---|---|---|---|---|---|---|---|---|---|
| **a. Standard** | | | | | | | | | | | |
| | Primary Visual | 0.20 | 4.54 | -27 | -92 | -18 | 0.23 | 4.24 | 30 | -97 | -10 |
| | pSTS | 0.13 | 3.16 | -41 | -60 | 31 | 0.15 | 3.04 | 43 | -44 | 7 |
| | mSTS | - | - | - | - | - | 0.27 | 3.79 | 61 | -32 | 4 |
| | aSTS | 0.24 | 3.65 | -55 | 8 | -14 | - | - | - | - | - |
| | Extrastriate | 0.15 | 2.94 | -53 | -62 | 7 | - | - | - | - | - |
| | TPJ | 0.14 | 2.77 | -55 | -49 | 26 | 0.13 | 2.94 | 52 | -50 | 27 |
| | Precuneus | 0.18 | 4.20 | -9 | -61 | 73 | 0.18 | 3.69 | 8 | -59 | 53 |
| **b. Controlling for attention** | | | $t_{(28.12)}$ | | | | | $t_{(28.12)}$ | | | |
| | Primary Visual | 0.22 | 5.22 | -27 | -92 | -18 | 0.20 | 3.67 | 30 | -97 | -10 |
| | pSTS | 0.10 | 2.79 | -40 | -56 | 27 | 0.14 | 2.84 | 44 | -45 | 6 |
| | mSTS | - | - | - | - | - | 0.25 | 3.73 | 61 | -32 | 4 |
| | TPJ | 0.11 | 3.26 | -61 | -43 | 30 | 0.14 | 3.06 | 45 | -60 | 33 |
| | Precuneus | - | - | - | - | - | 0.13 | 2.59 | 9 | -58 | 54 |

**Supplementary Table S3:** *Peak coordinates from Adult versus Child low-motion contrast from Supplementary Figure S3.*

Note: All Pearson *r* values were normalized for group analysis and then inverse transformed (*z* to *r*) back into correlation values. All coordinates are in MNI space and were projected into volumetric space using a spatially normalized surface mesh. pSTS, posterior superior temporal sulcus; mSTS, middle superior temporal sulcus, aSTS, anterior superior temporal sulcus; TPJ, temporoparietal junction.

| | Region | Left Hemisphere r | Left Hemisphere t(62.13) | x | y | z | Right Hemisphere r | Right Hemisphere t(62.13) | x | y | z |
|---|---|---|---|---|---|---|---|---|---|---|---|
| **a. p < 0.05** | | | | | | | | | | | |
| | pSTS | - | - | - | - | - | 0.03 | 4.00 | 49 | -62 | 24 |
| | mSTS | 0.03 | 2.77 | -46 | -33 | 0 | 0.04 | 2.94 | 44 | -45 | 4 |
| | TPJ | 0.03 | 4.45 | -47 | -55 | 20 | 0.03 | 4.86 | 55 | -45 | 25 |
| | Extrastriate | 0.04 | 3.38 | -50 | -78 | 7 | 0.04 | 2.72 | 44 | -76 | 25 |
| | Precuneus | 0.03 | 2.95 | -12 | -53 | 57 | 0.03 | 3.18 | 4 | -51 | 61 |
| **b. p < 0.01** | | | *t(62.13)* | | | | | *t(62.13)* | | | |
| | TPJ | 0.03 | 4.45 | -47 | -55 | 20 | 0.03 | 4.86 | 55 | -45 | 25 |
| | Precuneus | 0.03 | 2.95 | -12 | -53 | 57 | 0.03 | 3.18 | 4 | -51 | 61 |

**Supplementary Table S4:** *Peak coordinates from traditional analysis of the relationship between Child-to-Adult ISC age in our standard preprocessing from Supplementary Figure S5.*

Note: All Pearson *r* values were normalized for group analysis and then inverse transformed (*z* to *r*) back into correlation values. All coordinates are in MNI space and were projected into volumetric space using a spatially normalized surface mesh. pSTS, posterior superior temporal sulcus; mSTS, middle superior temporal sulcus; TPJ, temporoparietal junction.

| | Region | Left Hemisphere *r* | *t*(62.13) | *x* | *y* | *z* | Right Hemisphere *r* | *t*(62.13) | *x* | *y* | *z* |
|---|---|---|---|---|---|---|---|---|---|---|---|
| **a. p < 0.05** | | | | | | | | | | | |
| | pSTS | - | - | - | - | - | 0.03 | 3.26 | 49 | -62 | 24 |
| | mSTS | 0.02 | 4.07 | -67 | -47 | -13 | 0.03 | 2.56 | 44 | -45 | 4 |
| | TPJ | 0.03 | 3.77 | -47 | -55 | 20 | 0.03 | 3.79 | 58 | -46 | 23 |
| | Precuneus | 0.03 | 4.04 | -5 | -64 | 27 | 0.02 | 3.30 | 8 | -60 | 46 |
| **b. p < 0.01** | | | *t*(62.13) | | | | | *t*(62.13) | | | |
| | TPJ | 0.03 | 3.77 | -47 | -55 | 20 | - | - | - | - | - |
| | Precuneus | - | - | - | - | - | 0.02 | 3.30 | 8 | -60 | 46 |

**Supplementary Table S5:** *Peak coordinates from traditional analysis of the relationship between Child-to-Adult inter-SC and age while controlling for attention from Supplementary Figure S5.*

Note: All Pearson *r* values were normalized for group analysis and then inverse transformed (*z* to *r*) back into correlation values. All coordinates are in MNI space and were projected into volumetric space using a spatially normalized surface mesh. pSTS, posterior superior temporal sulcus; mSTS, middle superior temporal sulcus; TPJ, temporoparietal junction.

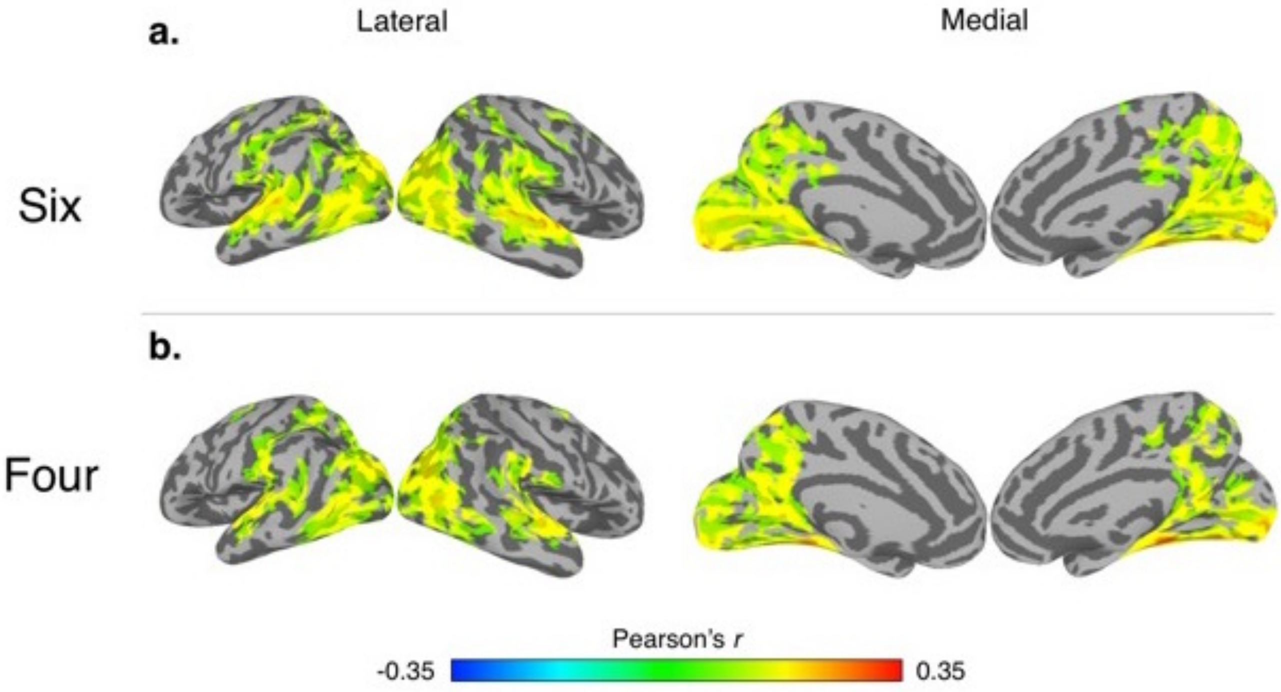


**Supplementary Figure S1. Within-group neural synchrony.** Within-group inter-SC maps for the (A) 6-year old and (B) 4-year old groups. Maps are thresholded at a nodewise $p > 0.01$ with a cluster extent of $315mm^2$ to reflect a FWE of $p < 0.05$.

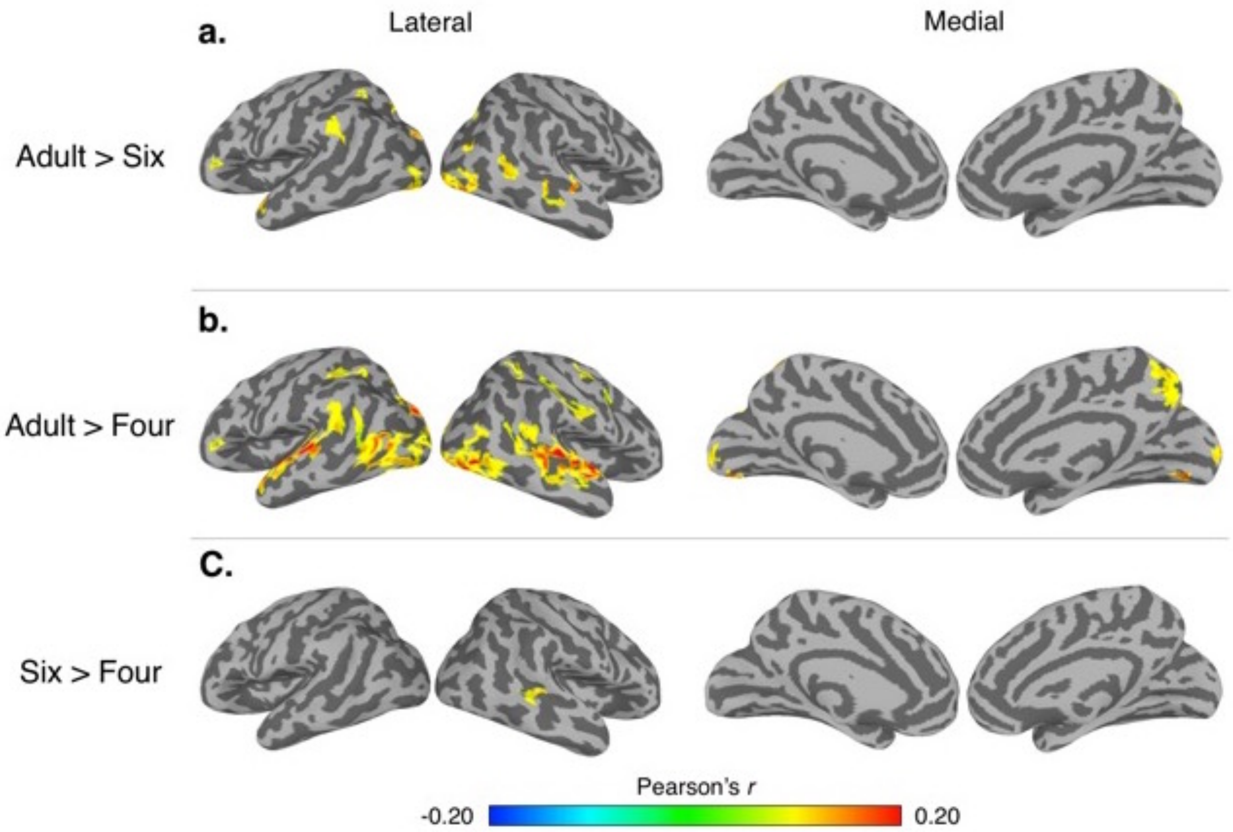


**Supplementary Figure S2. Separate between-group contrasts.** A between-group contrasts were made for (A) Adult vs. Six, (B) Adult vs. Four, and (C) Six vs. Four groups. Maps are thresholded at a nodewise $p > 0.05$ with a cluster extent of $150mm^2$.

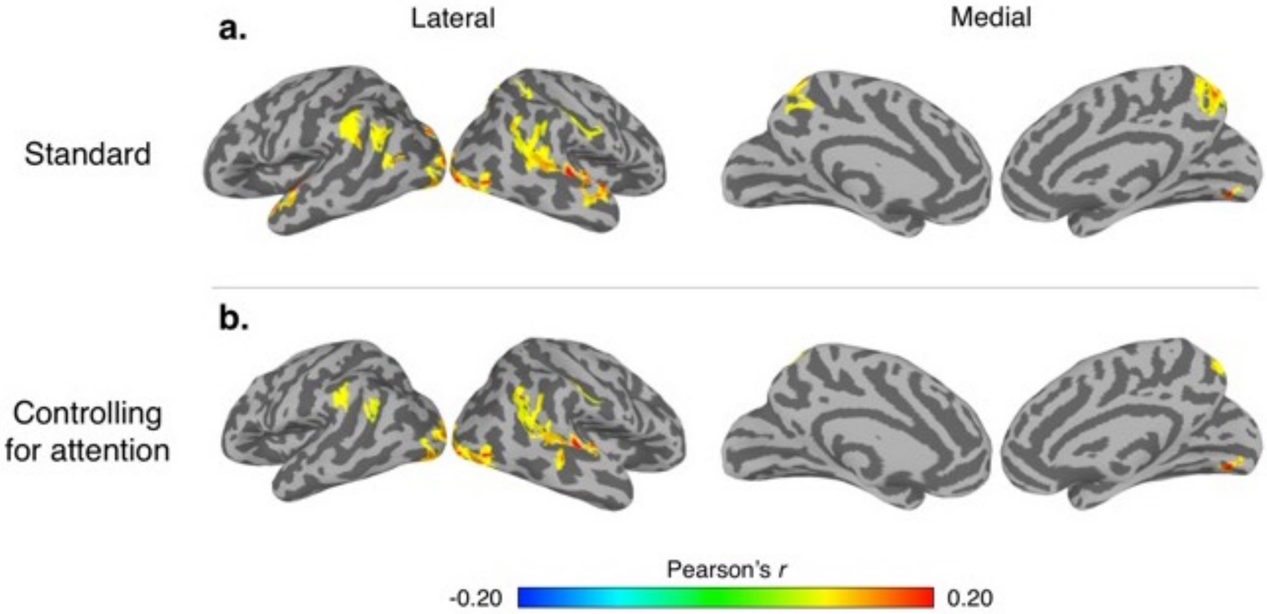


**Supplementary Figure 3. Adult vs. Child in low-motion sample (FD<.15 mm).** (A) Adult vs. Child contrast for the standard preprocessing with a low-motion threshold and (B) when controlling for attention with a low-motion threshold. Maps are thresholded at a nodewise $p > 0.05$ with a cluster extent of 150mm$^2$, uncorrected.

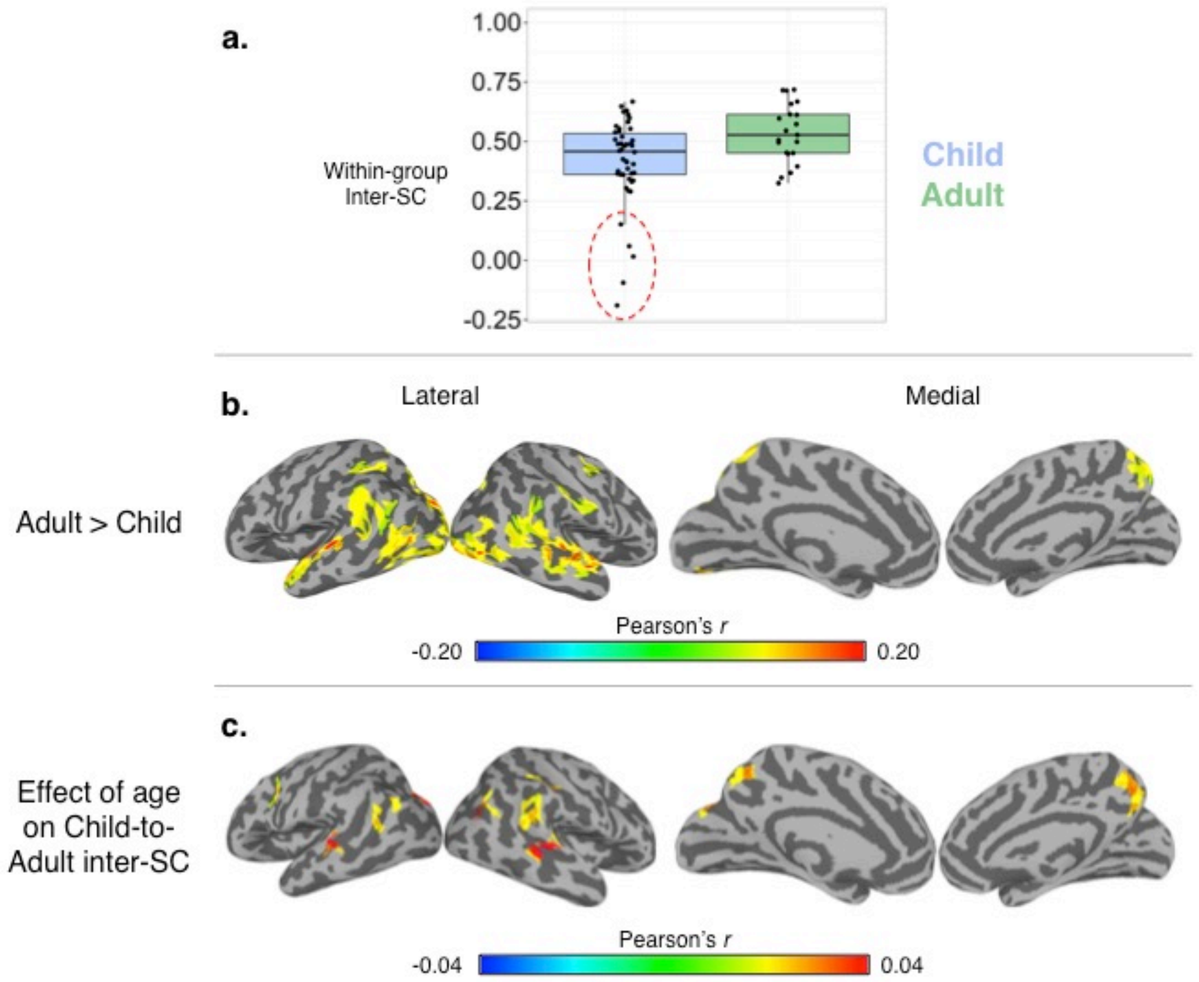


**Supplementary Figure 4. FEF outliers**. (a) Average within-group inter-SC within the FEF ROI for the Child and Adult groups. Outliers detected using the median absolute deviation are circled in red. (b) Adult vs. Child within-group inter-SC after removing the five FEF outliers. Nodewise threshold of $p < 0.05$, with a cluster extent of 315mm$^2$, corrected to a FWE of $p < 0.05$. (c) Child-to-Adult inter-SC as a function of age after removing the FEF outliers. Nodewise threshold of $p < 0.05$, with a cluster extent of 150 mm$^2$, uncorrected.

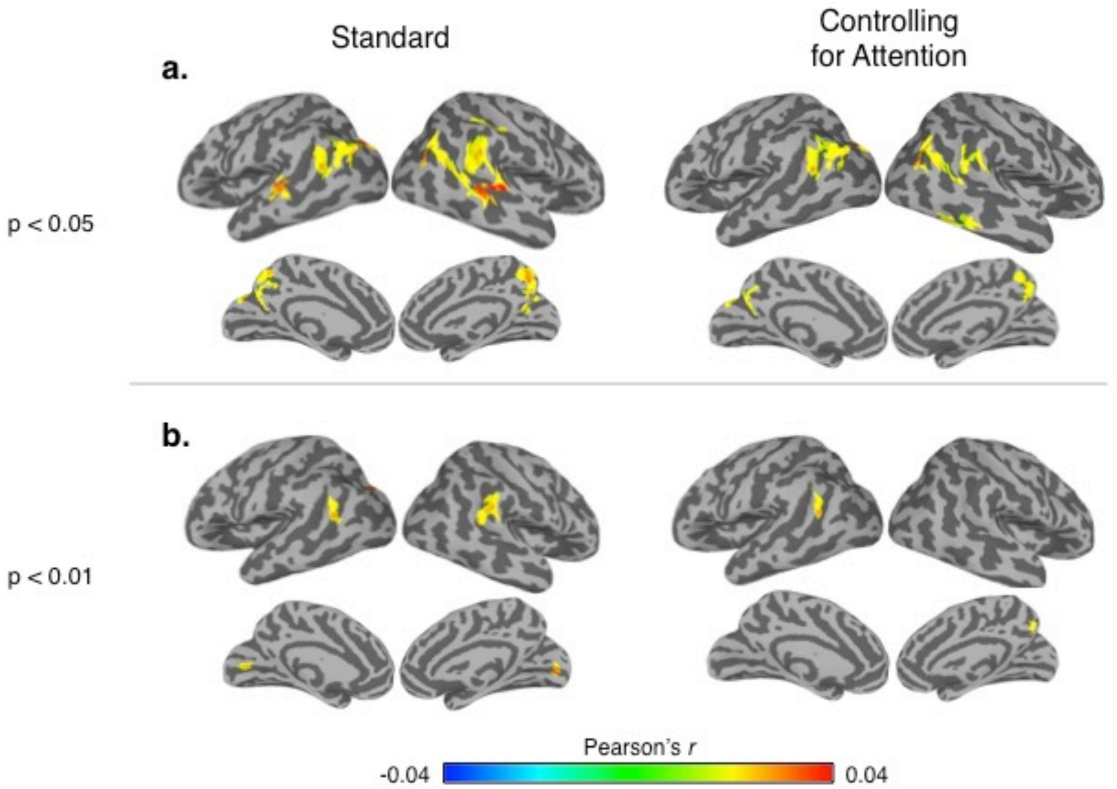


**Supplementary Figure 5. Traditional analysis of the relationship between Child-to-Adult inter-SC and age.** We use child age (in months) to predict average Child-to-Aduld inter-SC in our standard preprocessing and while controlling for attention. (a) Nodewise threshold of $p < 0.05$ with a cluster extent of $315mm^2$ and (b) Nodewise threshold of $p < 0.01$ with a cluster extent of $150mm^2$. All maps thresholded to reflect a FWE of $p < 0.05$.
